# Visual Versus Digital Color Determination of 3D-Printed Teeth as an Exercise in Dental Students’ Education

**DOI:** 10.3390/dj12020024

**Published:** 2024-01-26

**Authors:** Elisabeth Prause, Wolfgang B. Hannak, Robert Nicic, Holger A. Jakstat, Klaus Böning, Thomas Klinke

**Affiliations:** 1 Department of Prosthodontics, Geriatric Dentistry and Craniomandibular Disorders, Charité-Universitätsmedizin Berlin, Corporate Member of Freie Universität Berlin and Humboldt-Universität zu Berlin, 14197 Berlin, Germany; wolfgang.hannak@charite.de (W.B.H.); robert.nicic@charite.de (R.N.); 2 Department of Prosthodontics and Material Science, University of Leipzig, 20251 Leipzig, Germany; holger.jakstat@medizin.uni-leipzig.de; 3 Department of Prosthetic Dentistry, Faculty of Medicine Carl Gustav Carus, Technische Universität Dresden, 01069 Dresden, Germany; klaus.boening@uniklinikum-dresden.de; 4 Department of Prosthodontics, Gerodontology and Dental Materials, Center of Oral Health, University Medicine Greifswald, 17489 Greifswald, Germany; klinke@uni-greifswald.de

**Keywords:** color determination, shade deviation, 3D printing, education, spectrophotometer, dentistry

## Abstract

Visual color determination is part of the daily routine in dental practice. However, it is not a part of dental education so far. The aim of this study was to evaluate whether visual or digital tooth color determination of 3D-printed teeth is a reliable tool for inexperienced dentistry students. Preclinical dental students evaluated eleven 3D-printed, tooth-shaped samples (VarseoSmile Crown plus, BEGO, Bremen, Germany) of different color shades. Visual shade determination using a reference scale (3D-Master Toothguide (3DM_TG), VITA Zahnfabrik, Bad Säckingen, Germany), followed by a digital color determination using a spectrophotometer (VITA Easyshade V, (ES_V), VITA Zahnfabrik), was performed. Color deviation was calculated in the Lab* color space (ΔE00) and converted into CIELAB 2000. The results were evaluated using the Mann–Whitney U test and the Wilcoxon Rank Sum test (α = 0.05). Significant differences between visual and digital color determination were proven (*p* < 0.001). Visual color determination (3DM_TG) showed a mean deviation (ΔE00 ± 95%CI) of 6.49 ± 0.47. Digital color determination (ES_V) showed significantly lower mean deviations of ΔE00 of 1.44 ± 0.58. Digital tooth color measurement using a spectrophotometer was a more reliable tool for the color determination of 3D-printed teeth for inexperienced dentistry students.

## 1. Introduction

Nowadays, esthetics and tooth color play an important role in dentistry [1]. This is mainly due to an increased demand from patients for highly esthetic restorations [2,3,4]. Therefore, the determination of tooth color plays a decisive role with regard to a successful and satisfactory treatment [5].

Since digital dentistry is coming into focus, computer-aided design/computer-aided manufacturing (CAD/CAM) plays an important role in today’s dental practice. Various new CAD/CAM hybrid materials for additive (AM) and subtractive manufacturing (SM) have appeared on the dental market [6]. Three-dimensional-printable CAD/CAM hybrid dental materials are increasingly coming to the fore. Today, several CAD/CAM hybrid 3D-printable materials for long-term provisional restorations are available on the dental market. So far, one CAD/CAM hybrid material for additive manufacturing is approved for permanent single-tooth restorations (VarseoSmile Crown plus, BEGO, Bremen, Germany) according to the Medical Device Regulation as a class IIa material. This material can be printed by digital light processing (DLP). DLP is considered one of the most widely applied 3D-printing technologies for dental applications [7]. DLP features more economical material consumption and an even more efficient digital workflow than subtractive manufacturing [8]. The color stability of CAD/CAM hybrid 3D-printed materials and restorations is largely unknown. According to Espinar et al., the color and optical properties of CAD/CAM hybrid 3D-printed restorations have not been sufficiently evaluated in scientific studies so far [9].

In general, tooth color can be determined visually or digitally [5]. So far, only few scientific studies on CAD/CAM hybrid 3D-printable materials are available. The influence of color stability is largely unknown. Espinar et al. confirmed that the optical properties of CAD/CAM hybrid 3D-printed restorations depend on thickness and building orientation [10]. Current in vitro studies on CAD/CAM hybrid 3D-printed restorations are mainly limited to static tests evaluating intrinsic material properties, such as material strength [11,12,13,14,15,16,17,18,19,20], resilience [16], fracture toughness [16,19], microhardness [11,21,22], wear [23], surface roughness [18,22,24], and modulus of elasticity [11,17]. No studies are available regarding the color determination of CAD/CAM hybrid 3D-printed teeth.

It is known that individual color perception results in high variability in the visual determination of tooth color [25,26,27]. The insufficient standardization of lighting also contributes to high variations in color determination [28]. Consequently, inhomogeneous results in visual color determination are produced [28]. Furthermore, it is known that color perception also depends on age, gender, education, and profession [29,30]. Regarding gender, studies show that men and women differ in their capacity to distinguish shades [31,32]. In recent years, digital color measuring devices have, therefore, increasingly entered the dental market to ensure standardized color determination [33,34].

Regarding digital color determination, spectrophotometers are described as the most beneficial and convenient devices for color determination and are, therefore, widely used for this purpose [29,35]. Color determination using spectrophotometers is expected to be a reliable and objective method [29,36,37] compared with visual color determination. However, there is still no agreement about the ideal color determination method [4,29,38,39,40]. Some studies stated that a combination of visual and digital would be beneficial and lead to better results [29,35,41]. Mostly, digital analysis, with the help of spectrophotometers, has shown better results [42,43,44]. Furthermore, the use of a dental spectrophotometer showed a significantly higher percentage of correct shade matches compared with visual shade matching [45]. Furthermore, studies reported on the high repeatability and high reproducibility of digital color measuring devices [33,34].

So far, no scientific study regarding the visual versus digital color determination of 3D-printed teeth is available. Furthermore, no data are available regarding a direct comparison of color determination by dentistry students. The present study is, therefore, first to regard appropriate and reliable color measurement results during education. Additionally, the present study offers the first results about color values of a newly available 3D-printable material which will be one of the future hybrid materials in dentistry. Since color determination and esthetics are key factors to clinical success, establishing a method of determining the color of each material is a decisive step in improving everyday clinical dental routine.

The aim of the present study was to compare the results of visual and digital tooth color determination of 3D-printed teeth conducted by inexperienced dentistry students. The null hypothesis is that there is no difference in the results of visual and digital tooth color determination.

## 2. Materials and Methods

**Figure 1:**
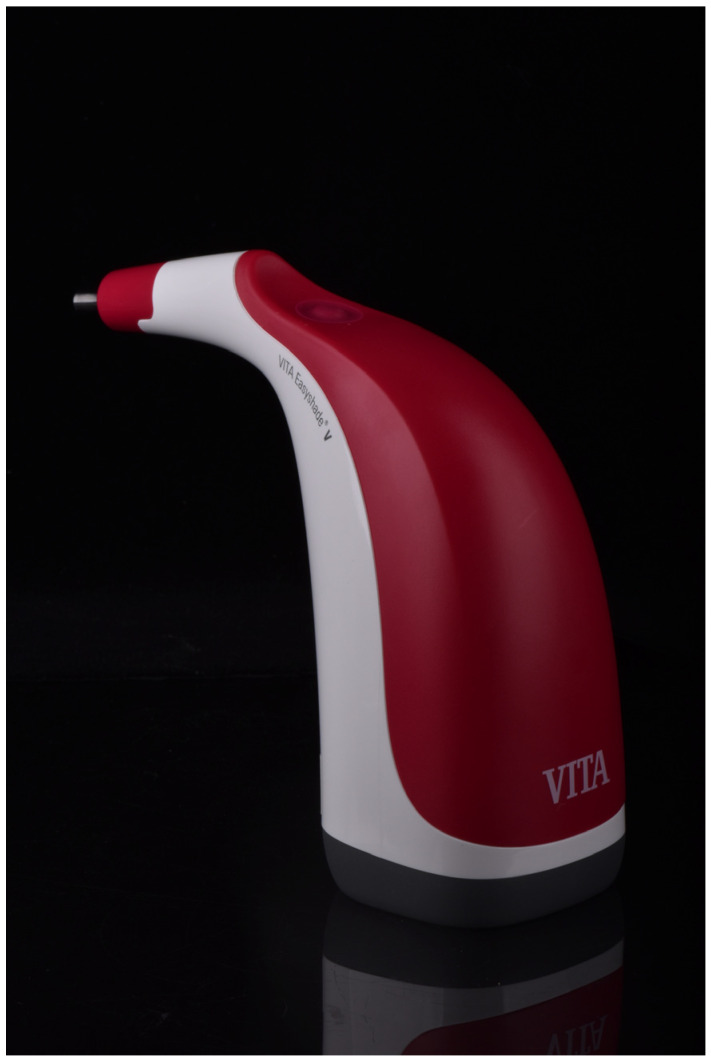
In the present study, a spectrophotometer (Vita Easyshade V, Vita Zahnfabrik) was used for digital color determination [1].

Dental students (female *n* = 28, male *n* = 10) in the preclinical and clinical semesters of the University of Leipzig, the University of Dresden, the Charité Berlin, and the University of Greifswald voluntarily participated in this study. The mean age was 23.5 ± 2.65 years, with a median of 23.0 ± 13.0 years. The dentistry students were from the second and third academic years. The second year belongs to the preclinical part, whereas the third year already belongs to the clinical part of dentistry studies. No pre-training or pre-calibration was conducted. This study was approved by an ethics committee at the University of Greifswald (BB 175/22). The participants evaluated 11 3D-printed tooth-shaped specimens (VarseoSmile Crown plus, BEGO, Bremen, Germany) (Table 1) of different color shades (A1, B1, A2, A3, B3, D3, and C2). Additional shade samples (B4Ϯ, C3¥, A4θ, and C4†) were fabricated by staining the 3D-printed dentin color of the specimens (GC Optiglaze color and Optiglaze, GC Corp., Tokyo, Japan): B3Ϯ, C2¥,†, and A3,5θ (Figure 1).

**Figure 2:**
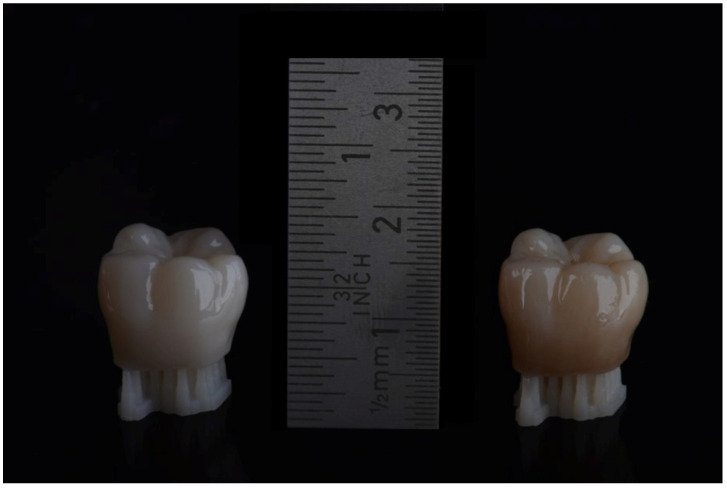
Three-dimensional-printed teeth of different color shades.

Visual and a digital color determination were conducted by each participant for each specimen. For visual color matching, a reference scale (3D-Master Toothguide (3DM_TG), Vita Zahnfabrik, Bad Säckingen, Germany) [47] was used, while the digital color determination was conducted with the help of a spectrophotometer (Vita Easyshade V (ES_V), Vita Zahnfabrik) [1] (Figure 1 and Figure 2). Visual color determination was conducted in dental treatment rooms under constant natural light at lunchtime, supported by room lighting. Digital color determination was also carried out at the same time and the same conditions as described above. An average measurement was carried out. For this purpose, the same area of the 3D-printed tooth was measured three times, and the average value and the corresponding tooth shade were recorded.

The evaluation included assessing the color differences of the templates compared to the chosen pattern using the L* a*b* color space, which is a color model representing colors in three dimensions: L* (lightness), a* (green to red), and b* (blue to yellow). The L*, a*, and b* values in the CIELAB color space are used to describe color in a systematic way. Additionally, the data were converted into the CIELAB 2000 color space [48].

Sharma et al. provided a method for transferring and calculating color differences based on the CIEDE2000 formula (ΔE00). The CIEDE2000 formula is an advanced color-difference formula that aims to improve upon previous models by addressing certain perceptual inconsistencies. It takes into account factors such as lightness, chroma, and hue differences [48].

The evaluation of the color differences was based on the recommendations of the Commission Internationale de l´Eclairage using the following formula [49]:

(1) $$\Delta E\equiv \sqrt{{\left(L1-L2\right)}^{2}+{\left(a1-a2\right)}^{2}+{\left(b1-b2\right)}^{2}}$$

Sharma et al. reported on how to calculate color differences between two colors in a way that aligns better with human perception according to the CIEDE2000 formula (ΔE00) [48]. The already-mentioned formula was developed in 2001 and was considered to improve the existing CIELAB formula. Both formulas calculate color differences, but the CIEDE 2000 formula seemed to be more useful in the clinical context [50].

In order to perceive color differences (ΔE00), the following formulas have to be taken into account.

The Euclidean distance formula is a common method for calculating the straight-line distance between two points in a multidimensional space. In the context of color-difference calculations in the L* a*b* color space, it appears that the formula is being used to determine the distance between two colors represented by their coordinates (L1, a1, and b1) and (L2, a2, and b2) (2) (3):

(2) $$\Delta E_{ab}=\sqrt{{\left(L1-L2\right)}^{2}+{\left(a1-a2\right)}^{2}+{\left(b1-b2\right)}^{2}}$$

(3) $$\Delta E_{ab}=\sqrt{\Delta L^{2}+\Delta a^{2}+\Delta b^{2}}$$

The distance (ΔEab) was calculated using the above-mentioned Euclidean formula (3). Once the color differences (ΔEab) were calculated for each student, the results were summarized, and the mean average ΔEab value was calculated separately for both groups.

The changes in the total color differences for each student regarding visual and digital color determination represented the target variable. The results were evaluated using a statistics program (Sigmastat 13, Sysstat, Palo Alto, CA, USA) with non-parametric, rank-scaled methods using median and the 25% and 75% quartile. The significance level was set to α = 0.05.

## 3. Results

**Figure 3:**
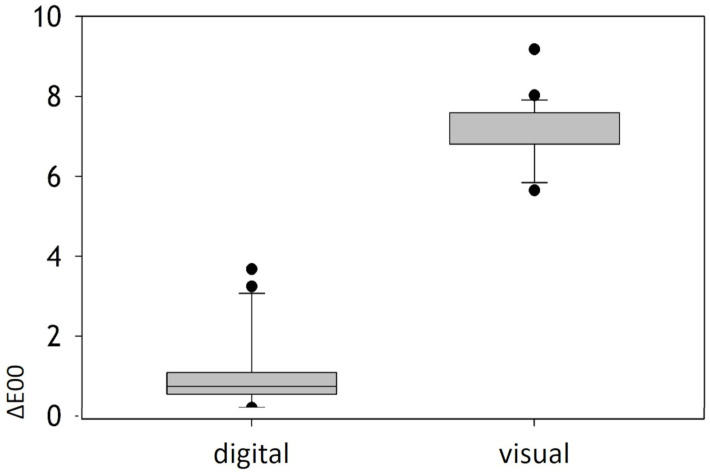
Comparison between visual and digital color determination. The ΔE00 values were significantly higher than ΔE00 values for digital color determination.

After correcting the erroneous documentations, the color deviation (ΔE00) of the templates’ colors was calculated as a function of the evaluation method (digital vs. visual). For visual color matching using 3DM_TG, a mean deviation (ΔE00 ± 95%CI) of 6.49 ± 0.47 was recorded. The deviations were significantly lower (*p* < 0.001) in the ES_V color control group, with a mean ΔE00 of 1.44 ± 0.58. In the digital color discrimination group, the median (±interquartile range) was 0.92 ± 0.21, which was significantly lower than the median of the digital visual color discrimination control group at 6.59 ± 0.77 (*p* < 0.001). In the visual color determination group, the standard deviation and standard error (±SD/SE) were ±1.15/0.22, whereas in the digital color verification group, they were ±1.30/0.28. Significant differences between the two groups (3DM_TG vs. ES_V) were observed at the *p* < 0.001 level (Figure 3). The deviations of the visual color determination were larger, while the results of the digital color determination were below the perceptibility thresholds.

**Figure 4:**
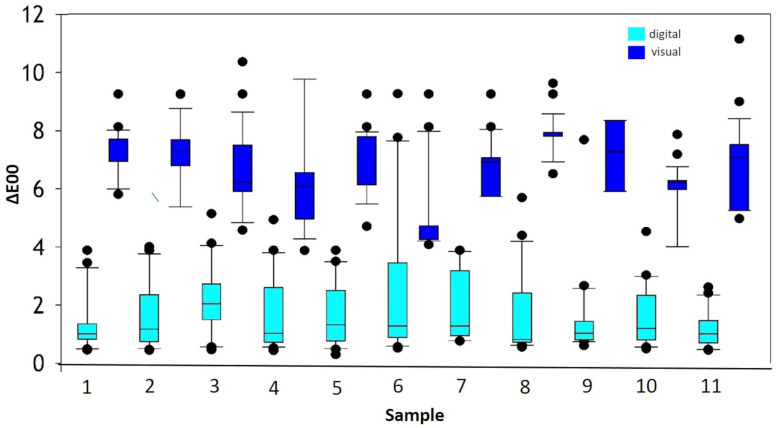
All samples showed ΔE00 values that were higher for visual color determination than for digital color determination.

When arranging the randomized presented samples by value, in descending order of template brightness, no significant differences were found between the observation groups (*p* > 0.05). The level of experience of the dentistry students did not play a role regarding the results of visual and digital color determination. The brightness of the templates did not have an influence on color matching (see Figure 4).

## 4. Discussion

The aim of the present study was to compare the results of visual and digital tooth color determination on CAD/CAM hybrid 3D-printed teeth conducted by inexperienced dentistry students. The results showed that visual tooth color determination, compared with digital tooth color determination, is an insufficient method to evaluate the color deviations of CAD/CAM hybrid 3D-printed teeth conducted by inexperienced dentistry students. The null hypothesis must be rejected.

The color-shade deviations of the visual tooth color determination were outside of the limit of perception, whereas the results of the digital tooth color determination were below the limit of perception. The mean deviation was 6.49 ± 0.47 for visual color determination, and the deviations of the results of digital determination were 1.44 ± 0.58. Rade et al. reported a 50%/50% perceptibility threshold of ΔE00 = 0.8 and a 50:50% acceptance threshold of ΔE00 = 1.8 [51].

Earlier studies already investigated visual and digital color determination. Most studies stated that digital analysis using spectrophotometers showed better results [42,43]. Digital measuring devices are considered to improve the accuracy of shade matching as well as the interpretation and fabrication of dental restorations [42,43]. Furthermore, spectrophotometers are, therefore, described as the most beneficial and convenient devices for color determination and are, therefore, widely used for this purpose [29,35]. Color determination using spectrophotometers is expected to be a reliable and objective method [29,36,37].

Since color stability and its influencing factors of CAD/CAM hybrid 3D-printed materials are not completely scientifically evaluated, the present study showed that digital color determination led to more reliable results. The reason might be that digital color determination is conducted in a specific moment without any possibility of color change.

These results could be proven in the present study. Since only reduced data regarding CAD/CAM hybrid 3D-printed materials and their color properties are available so far, the present study offers the first results regarding CAD/CAM hybrid 3D-printed materials.

A review conducted by Espinar et al. concentrated on the color and optical properties of CAD/CAM hybrid 3D-printed materials [9]. The review showed that the included studies did not report on the color coordinates of the tested 3D-printed materials or their agreement with a dental shade guide [9]. Furthermore, no information was given regarding the printing process, which could influence the color properties of the final 3D-printed restorations a lot [9]. However, the included studies primarily used clinical spectrophotometers or a colorimeter. The CIELAB color space was used for an evaluation of color. These methods were comparable to the present study, where a spectrophotometer and the CIELAB color space were taken into account. Still, it was mentioned that different surface treatments and post-curing time could have an enormous influence on the color stability of CAD/CAM hybrid 3D-printed restorations [9]. Consequently, the influencing factors on color stability and color perception of CAD/CAM hybrid 3D-printed materials have not been adequately scientifically evaluated [9]. Future studies, such as the present one, were recommended regarding the printing conditions [9]. However, for improving the clinical effectiveness of CAD/CAM hybrid 3D-printed restorations, it is essential that the optical behavior be understood [10]. Since Espinar et al. showed that the color perception of CAD/CAM hybrid 3D-printed materials depends on thickness and building orientation, it could be influenced a lot by these factors, especially for inexperienced dentistry students. It could be proven that the scattering of light in 3D-printed materials is one of the most relevant light attenuations [10]. Furthermore, the scattering, absorption, and transmittance of different CAD/CAM hybrid 3D-printed materials were similar. Due to its liquid character, the distribution of fillers might be inhomogeneous. However, the amount of filler has an influence on the mechanical stability of restorations but not directly on color stability. The filler distribution might have an influence on color perception since it is known that variations in translucency were related to differences in crystal volume and the scattering of light in all-ceramic materials [52]. Less scattering of light can be achieved with a lower crystalline content. Therefore, translucency can be influenced by the crystal volume [52]. However, color perception and determination could be influenced a lot by the scattering of light and the filler distribution of CAD/CAM hybrid 3D-printed restorations. Consequently, future studies are necessary to analyze the filler distribution before and after the 3D-printing process. Furthermore, microstructural analyses would be beneficial to evaluating the influence of the microstructure, color perception, and stability of CAD/CAM hybrid 3D-printed materials in vitro and in vivo. In general, digital color determination seems to be an independent tool, especially for dentistry students when conducting color measurements of CAD/CAM hybrid 3D-printed restorations. The results of the present study could, therefore, be beneficial for upcoming clinical studies using CAD/CAM hybrid 3D-printed restorations. The clinically relevant thicknesses of restorations and building orientations based on light reflectivity should be paid attention to in order to improve the clinical outcome of these materials [10].

So far, color determination has not been routinely included in the curriculum. However, it has already been shown that routinely given non-specific color training for dentistry students regarding color determination was evaluated positively [29]. The present study, therefore, proved that digital color determination with the help of a spectrophotometer led to higher reproducibility among inexperienced dental students. These results were also confirmed by other studies [29,53,54]. They described a high measurement accuracy of 92.6% and repeatability [29,55] of 96.4%. The advantage of a spectrophotometer is that it is not dependent on environmental light conditions [29,56], but it generates its own light [29]. Still, it has to be mentioned that digital color determination could also be influenced by varying the angles to the tooth while positioning the measurement head and differences in reflection processing. Moreover, the contact pressure might influence the measuring results [57].

Students with clinical experience tend to be more successful with color determination [29]. Furthermore, color determination could be improved by giving training to dentistry students [5,29,58]. Still, studies showed that the level of experience has a high influence on correct clinical color determination [28,31,59,60]. However, studies exist that proved that there was no positive correlation of the level of experience and the capacity for color determination [31,61,62,63,64]. Curd et al. stated that experience did not have an influence on the color determination of dentistry students [65]. Compared with other dental students´ education methodology, the main goal of all these studies is to prepare dentistry students effectively for clinical practice [66,67]. In this context, adequate teaching resources are mandatory. Digital dentistry especially has been developing enormously within the last decades. Therefore, students have to be educated and well prepared for modern technology. Next to newly available digital devices, as described in the present study for the digital color determination of teeth, other new technologies gain importance in transmitting knowledge and facilitating the acquisition of skills [66]. Additionally, the current generations of students are open-minded and familiar with digital technology [66]. Llena et al. evaluated the efficacy of augmented reality in dentistry students gaining knowledge and skills among when designing and analyzing cavity preparations [66]. The results showed that the augmented reality technique favored the gaining of knowledge and skills and was evaluated as a useful tool by the students [66]. Furthermore, studies showed that color determination and selection could be influenced by gender [31]. Since color blindness is a hereditary disorder that is connected to the X chromosome, men tend to have a 10- to 20-times-higher risk for this deficiency [31,68]. People suffering from color deficiency have difficulties differentiating differences in chroma and hue [61,65]. However, it could be shown that women were not always more successful in color determination than men [31]. Furthermore, Moser et al. evaluated a group of 670 dentists and confirmed that 10% had a deficiency related to color perception [31,69]. Basically, women are considered to achieve better results regarding color determination and color perception than men [31,32,70,71]. Since studies exist that showed that there was no difference regarding gender in the ability to determine and select color [31,64], the influence of gender has to be considered critically. The present study did not distinguish between the color determination results of women and those of men. Therefore, no correlation can be drawn about the influence of gender on color determination.

Another influencing factor regarding color perception and color determination is the used light source. It is known that dentists are inconsistent regarding color matching [39,65,72,73]. Furthermore, it was shown that some dentists changed their color selection every day [65,73]. Consequently, a consistent light source would be beneficial for reliable color determination [65]. Curd et al. analyzed the effect of a light-correcting device and its effect on color determination of color-deficient dentistry students [65]. The results of color-deficient students differed from those of non-color-deficient students [65], especially when color selection was conducted under natural light. In contrast, color determination did not differ that much when a correcting light source was used [65]. Curd et al. concluded that a correcting light source under natural light led to better results regarding visual color determination by dentistry students [65]. In the present study, an additional light source, in the sense of room illumination, was used. Visual color determination was conducted at lunchtime to ensure sufficient natural light from outside. However, visual color determination led to more unreliable results compared with digital color determination in the present study. Again, it has to be mentioned that the advantage of digital color determination is that it is independent of the surrounding light and the background of the individual clinicians or students. Future studies should be performed comparing additional light sources when conducting visual color determination by students with and without color deficiency. A direct comparison with digital color determination also using spectrophotometers could be drawn. Since digital color determination seems to provide more consistent results due to optimized circumstances independent of the individual backgrounds of operators, the technology is not influenced by the experience, gender, or color deficiency of the operators. Therefore, it provides a reliable technique regarding color determination for all people working in the dental field. The present study is the first one to compare different methods of color determination on CAD/CAM hybrid 3D-printed teeth. However, this new materials class with so-far reduced scientific evaluation regarding color stability and color perception seemed to be evaluated better when digital color determination was conducted by dentistry students.

The CIEDE2000 was used in the present study for color determination evaluation since it has been described as the best possible evaluation method for small color differences [74]. Visible color differences at ΔE00 values of 2.6 and 3.7 could be shown in vivo [75,76]. The clinical acceptance was evaluated at ΔE values of 5.5 and 6.8 [75,76]. The CIEDE 2000 formula was developed in 2001 and was considered to improve the existing CIELAB formula. Both formulas calculate color differences, but the CIEDE 2000 formula seemed to be more useful in the clinical context [77] since it concentrates more on clinical perceptibility and acceptability. It is known that, in dentistry, the acceptability threshold of color difference is greater than the perceptibility threshold [76,78,79]. Moreover, in terms of lightness, chroma, and hue, the CIEDE 2000 formula provides an interactive term between chroma and hue differences for improving the performance of blue colors and a scaling factor for the CIELAB a* scale for improving the performance of grey colors [77]. It could be shown that the CIEDE 2000 formula reflected the color differences perceived by the human eye better than the CIELAB formula [80]. However, to obtain an ideal measuring position with an adequate contact area for the measurement head, in vivo conditions are often unlikely to be perfect regarding natural teeth [5]. Therefore, it is impossible to obtain the same measuring position each time in repeated measurements [5].

The results of the present study have to be taken with caution, since there were no restrictions regarding the measuring position and contact angle of the spectrophotometer. Furthermore, the present study is an in vitro study. Consequently, recommendations for clinical studies can hardly be derived. The number of participating dentistry students was small. It would be beneficial if the groups were bigger and more heterogeneous to ensure a proper statistical analysis. Furthermore, a precise distinction between the results of preclinical and clinical students should be performed in future studies, since these results are of great importance. Although the present study showed that a spectrophotometer provided reliable and more accurate data regarding the color values of newly available 3D-printable CAD/CAM hybrid materials, future studies about color consistency before and after the manufacturing process are desirable. Furthermore, a precise distinction between women and men is desirable. Additionally, different tools and spectrophotometers should be used next time to compare the color values in more detail. Therefore, the present study should be considered as a first step towards the reliable and appropriate color determination of newly available hybrid materials during education. It can be concluded that digital color determination is a reliable tool for dentistry students with less experience to achieve accurate results regarding color determination of CAD/CAM hybrid 3D-printed materials.

## 5. Conclusions

The present study showed that digital color determination is a reliable and manageable tool for inexperienced dentistry students when measuring newly available CAD/CAM hybrid 3D-printed teeth. The following conclusions can be derived from the results of the present study:

- ΔE00 values showed significant differences regarding visual and digital color determination when the measurements were conducted by more-or-less inexperienced dentistry students.
- The results of visual color determination can be significantly improved by the use of spectrophotometers.
- Digital color determination seems to be a reliable tool during the education of dentistry students.
- CAD/CAM hybrid 3D-printed teeth of different colors should be measured digitally regarding color determination.
- No statements can be made regarding the influence of gender.
- The level of experience of the dentistry students did not play a role regarding the results of visual and digital color determination.
- Further studies would be beneficial regarding the color stability of 3D-printable materials and evaluating other materials that are CAD-manufactured.

## Figures and Tables

**Table 1:** Information about the tested 3D-printed material with details regarding the composition [46].

Material	Composition	Manufacturer
VarseoSmile Crown plus	Esterification products of 4,4′-isopropylidiphenol, ethoxylated and 2-methylprop-2enoic acid, silanized dental glass, methyl benzoylformate, diphenyl (2,4,6-trimethylbenzoyl) phosphine oxide, 30–50 wt%—inorganic fillers; particle size 0.7 µm	BEGO, Bremen, Germany

## Data Availability

Raw data are available on request.
